# The oral microbiota and cardiometabolic health: A comprehensive review and emerging insights

**DOI:** 10.3389/fimmu.2022.1010368

**Published:** 2022-11-18

**Authors:** Yiwen Li, Mengmeng Zhu, Yanfei Liu, Binyu Luo, Jing Cui, Luqi Huang, Keji Chen, Yue Liu

**Affiliations:** ^1^ National Clinical Research Center for Chinese Medicine Cardiology, Xiyuan Hospital, Chinese Academy of Chinese Medical Sciences, Beijing, China; ^2^ The Second Department of Gerontology, Xiyuan Hospital, China Academy of Chinese Medical Sciences, Beijing, China; ^3^ China Center for Evidence-based Medicine of Traditional Chinese Medicine (TCM), China Academy of Chinese Medical Sciences, Beijing, China

**Keywords:** oral microbiome, cardiovascular disease, microbial metabolites, oral microbiota transplantation, periodontal disease

## Abstract

There is mounting evidence demonstrating that oral dysbiosis causes periodontal disease and promotes the development of cardiovascular disease. The advancement of omics techniques has driven the optimization of oral microbiota species analysis and has provided a deeper understanding of oral pathogenic bacteria. A bi-directional relationship exists between the oral microbiota and the host, and oral-gut microbiota transfer is known to alter the composition of the gut microbiota and may cause local metabolic disorders. Furthermore, cardiovascular health can also be highly affected by oral microbiota functions and metabolites, including short-chain fatty acids (SCFAs), nitric oxide (NO), hydrogen sulfide (H_2_S), and some lipid metabolites. Studies have found that trimethylamine oxide (TMAO) may have adverse effects on cardiovascular health, whereas SCFAs, NO, and H_2_S have cardioprotective effects. SCFAs and H_2_S exert varying oral and cardiovascular effects, however reports on this specific topic remain controversial. Previous evidences are accustomed to summarizing the functions of oral microbiota in the context of periodontitis. The direct relationship between oral microbiota and cardiovascular diseases is insufficient. By systematically summarizing the methods associated with oral microbiota transplantation (OMT), this review facilitates an investigation into the causal links between oral microbiota and cardiovascular disease. The concomitant development of omics, bioinformatics, bacterial culture techniques, and microbiota transplantation techniques is required to gain a deeper understanding of the relationship between oral microbiota and cardiovascular disease occurrence.

## 1 Introduction

With the advancement of multi-omics and bioinformatics, the microbe-host interactions in the human body have begun to gain an increasing amount of attention. There is mounting evidence to suggest that the commensal microbiota plays a crucial role in human health and disease development, including cardiovascular disease (1, 2). The oral microbiota is the second largest microbial community present in the human body (3). The ecological niches in the oral cavity are divided into the saliva, tongue, dental surface, gingiva, buccal mucosa, palate, and subgingival/supragingival sites, with variations in microbiota species and activity, as well as varying susceptibility to diseases across the different niches. The oral microbiota is dominated by *Streptococcus*, belonging to the *Firmicutes* phylum (36.7%) (4), which produces an abundance of primary and secondary metabolites, and is associated with the occurrence of systemic diseases (5). Studies have shown that age-related variations have little effect on the oral microbiota when compared to other habitats in the bodies of healthy populations (6, 7). Furthermore, new evidence suggests that oral microbiota are involved in the preliminary digestion of food in the oral cavity and produce a variety of metabolites (8, 9). Oral microbiota sampling is also highly convenient, and the tongue-coating morphology and dental plaques can be observed under direct vision. These advantages have therefore enabled the potentially rapid clinical translation of research on the oral microbiota.

The oral microbiota has immense potential and value with regard to research on cardiovascular disease, specifically atherosclerosis (10, 11). The bi-directional interaction between periodontal diseases and oral microbiota, as well as the interaction between periodontal diseases and cardiovascular disease, has long been investigated (12, 13). Periodontal diseases are associated with transparent pathogens of cardiovascular disease and are associated with hypertension, heart failure, atherosclerosis, and coronary heart disease (14–16). Aggressive treatment of periodontal disease can significantly reduce the risk of cardiovascular disease development (17).

Therefore, the oral microbiota may have a substantial impact on systemic disease. This review seeks to answer the following questions based on existing evidence: (1) How does the oral microbiota affect the progression of cardiovascular disease? (2) Does cardiovascular disease have a reciprocal effect on the oral microbiota? (3) What are the metabolites produced by the oral microbiota that affect cardiometabolic health? How do these metabolites regulate inflammation, oxidative stress, or vascular function? (4) Can the novel techniques and methods currently under investigation (such as oral microbiota transplantation [OMT]) be applied to research on oral microbiota? Our findings will potentially serve as a reference for future investigations on the relationship between oral microbiota and cardiovascular disease occurrence.

## 2 Oral dysbiosis and phenotypes of cardiovascular disease: Bi-directional causality

### 2.1 Periodontal diseases: The role of oral dysbiosis in cardiovascular disease

It has been reported that the presence of periodontal disease and dental plaque may exacerbate cardiovascular disease (18). Research conducted on the correlation between oral dysbiosis and cardiovascular disease is usually based on the effects of periodontal diseases (19, 20). Therefore, understanding the mechanism of these three comorbidities could be helpful and necessary.

Oral dysbiosis is a key feature of periodontitis and research has indicated that gram-negative bacterial populations are significantly increased in patients with periodontitis (21). The progression of periodontal inflammation is accompanied by community dysbiosis. 16S pyrosequencing and metagenomic sequencing results have indicated that the oral α**-**diversity was higher in patients with periodontitis when compared to healthy people (21–24) and this same trend has been observed in other systemic diseases; however, the correlation between oral α**-**diversity and cardiovascular diseases has still not been well researched (25). Studies have reported that specific key pathogens are significantly increased in patients with periodontal disease and atherosclerosis, such as *Streptococcus mutans* and *Porphyromonas gingivalis (* 26, 27). Clinical studies have reported that the density of the oral microbiota (in saliva, as well as in supra- and subgingival sites) was positively correlated with the severity of periodontal parameters, the number of periodontal pathogens, and severity of abdominal aortic aneurysm. These findings have been supported by serological and immunological studies, in which immunoglobulin G antibodies targeting *Porphyromonas gingivalis* and *Aggregatibacter actinomycetemcomitans* were detected in patient oral tissues (28).

Certain oral commensal bacteria that are found in coronary plaques are also present in non-cardiac organs, such as *Campylobacter rectus, Porphyromonas gingivalis, Porphyromonas endodontalis, Prevotella intermedia* and *Prevotella nigrescens* (29). It is yet to be established if these bacteria specifically influence the formation of atherosclerotic plaques or if it is an oportunistic infection. The pathogenic mechanism of periodontitis may help us understand the relationship between oral microbiota and cardiovascular diseases. It has been reported that cardiovascular disease may be caused by periodontal disease *via* mechanisms such as inflammatory response, oxidative stress, immune response, and platelet aggregation (19, 30). Variations in the manifestations of dysbiosis have also been observed between patients of different sexes and between patients with or without dental caries/missing teeth (31, 32). Studies have found that patterns of oral dysbiosis may induce the host’s immune response (30); however certain pathogens may synergistically induce immunosuppression through an association with signaling pathways. For example, pathogens may inhibit T helper 1 (TH1) cell-mediated immunity using complement Toll-like receptor regulation, which may disrupt functional receptor interactions (33), thereby aggravating the cardiovascular disease. Periodontal disease is an important marker of oral dysbiosis; however, it may not be the sole cause of cardiovascular disease. Oral dysbiosis leads to a host immune response (30), which exacerbates cardiovascular disease. Animal models of periodontitis include different approaches, one is surgical approach to periodontitis (34), and the others are associated with the oral microbiota (inoculation with microbial pathogens (35, 36) and lipopolysaccharide (LPS) injection (37)). Excluding the effect of surgical approach (34), oral microbiota or LPS could still promote systemic inflammation (38).

The relationship between oral microbiota and periodontal diseases is constantly evolving (39). A new hypothesis has recently emerged in microbial research suggesting that the onset of inflammatory or immune response may not be induced by a single type of pathogenic bacteria but by changes in the overall microbiota, and this idea has challenged the concept of oral pathobionts. Disrupted homeostasis may be associated with a series of upstream and downstream bacteria, rather than a specific low-abundance species (39). For example, the “red complex” bacteria (*Porphyromonas gingivalis, Treponema denticola*, and *Tannerella forsythia*) (40) were initially considered a major etiological factor of periodontitis; however, this perspective has now been challenged (41). This is because previous studies on oral microbiota using *in vitro* cultures may have overestimated the importance of bacterial species that are prone to growth, such as Gram-negative bacteria. However, more recent studies have found a significant increase in the abundance of Gram-positive anaerobic bacteria in the oral cavity of diseased individuals, sometimes even surpassing the abundance of Gram-negative bacteria (42). Omics research (43, 44) has verified that the pathogenesis of periodontitis involves a synergy and dysbiosis of multiple microorganisms, and is referred to as the polymicrobial synergy and dysbiosis model (45). Interestingly, the abundance of bacteria was negatively correlated with the relative abundance of *Porphyromonas ginglivlis.* In the low-abundance state, these typical pathogens promote the overall increase of the bacterial load, which can be indicated as a delicate ecological balance between mutualistic and antagonistic interactions in the microbiome (21).

### 2.2 The oral microbiota affects cardiometabolic health

**Figure 1:**
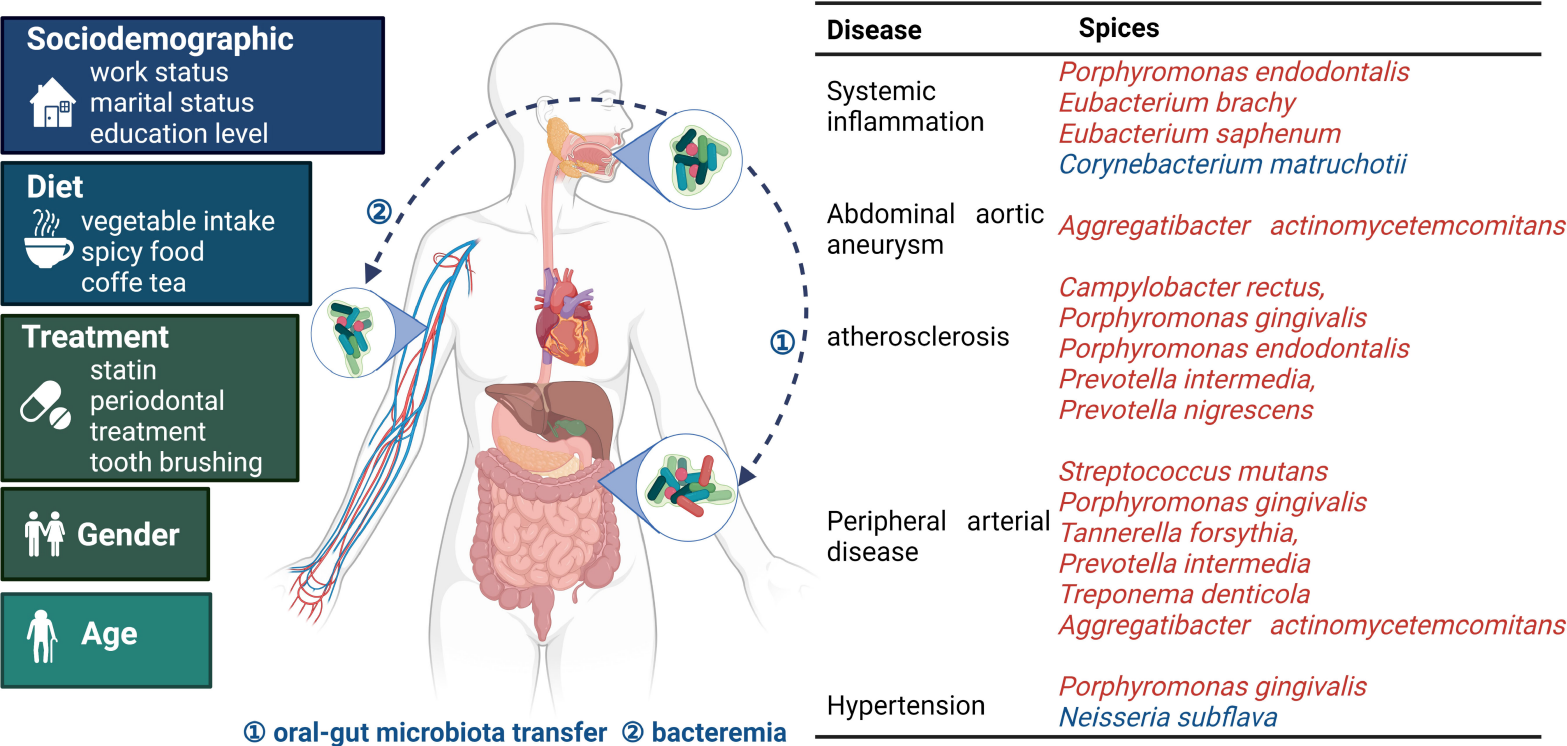
Oral microbiota and bacterial species related to cardiovascular disease occurrence The oral microbiota is influenced by multiple factors. Pathogens gain entry into systemic tissues *via* oral-gut microbiota transfer and bacteremia, thereby endangering cardiovascular health. The table presents the relationship between the microbiota and specific diseases. Red and blue indicate microorganisms with adverse and protective effects, respectively. Oral microbiota and cardiovascular disease common influencing factors are on the left (7, 18, 47, 48).

Oral dysbiosis is thought to be closely linked to cardiovascular disease and various species including, *Streptococcus mutans* and *Porphyromonas gingivalis*, which have been shown to increase with the occurrence of periodontal disease and systemic inflammation (26, 27). A number of other species, namely; *Treponema denticola, Tannerella forsythia, Prevotella intermedia, Prevotella nigrescens, Actictinobacillus actinomycetemcomitan, Campylobacter rectus, Parvimonas micra, Porphyromonas gingivalis*, *Porphyromonas endodontalis, Prevotella intermedia, Eubacterium timidum, Eubacterium brachy*, and *Eubacterium saphenum*, have also been found to be associated with oral dysbiosis and cardiovascular disease (29, 30, 46) **(** **Figure 1** **)**. As most of these reports were made after conducting cross-sectional studies, researchers were unable to determine the causal relationship between oral dysbiosis and periodontal disease occurrence (39).

The oral microbiota induces inflammatory and immune responses in oral tissues (49, 50), which in turn affects cardiometabolic health and promotes the onset of cardiovascular disease. The microbial invasion of the bloodstream (51) and alterations in gut microbiota caused by oral-gut microbiota transfer (52, 53) may also exacerbate systemic inflammation. Oral dysbiosis ultimately manifests as systemic inflammation, immunoreaction, oxidative stress, and thrombosis. Systemic inflammation may also disrupt the balance of the oral microbiota, as the relationship is bi-directional (54). This paper primarily focuses on discussing the potential upstream mechanisms of the abovementioned pathological outcomes (55).

#### 2.2.1 Crosstalk between the microbiota and local environment

Oral microbiota participates in the inflammation and immune regulation of local environment (56). Oral dysbiosis induces the recruitment of neutrophils and macrophages, which not only prevents further destruction of connective tissues by the microbiota, but also stimulates the immune responses of cells such as dendritic and gamma delta cells, thus inducing the release of pro-inflammatory mediators (tumor necrosis factor α (TNF-α); interleukin-1β (IL-1β); interleukin-17 (IL-17)), and regulates the function of T helper cells (21). It has been reported that these inflammatory states are positively correlated with the oral microbiota load, thereby creating a vicious cycle (21).

An elevated concentration of bacterial surface molecules, such as LPS or bacterial flagellins, stimulates the production of inflammatory mediators and cytokines, thereby promoting inflammation and immunoreaction (57, 58). The mechanisms of this stimulation may involve the activation of inflammatory pathways such as the matrix metalloproteinase 9 (MMP9) and Nuclear factor kappa-B (NF-κB) and Basic Helix-Loop-Helix ARNT Like 1 (BMAL1) pathways (27, 59). Under the effects of TNF-α, interleukin 6 (IL-6), and transforming growth factor β (TGFβ), epithelial and immune cells trigger the production of reactive oxygen species (ROS), reactive nitrogen species, and matrix metalloproteinases, which activate the NF-κB pathway (56).

These mechanisms may be involved in the formation of atherosclerotic lesions. In mice, *Porphyromonas gingivalis* infection induces the accumulation of macrophages and inflammatory mediators (such as CD40, interferon-γ (IFN-γ), IL-1β, IL-6, and TNF-α) in atherosclerotic lesions; however, the abovementioned inflammatory responses were shown to be milder in mice with congenital immunodeficiency (60). Animal experiments have demonstrated that *Porphyromonas gingivalis* and other bacteria can cause abnormal endothelial relaxation, and thus aggravate atherosclerosis and hypertension; however, periodontal treatment may improve endothelial function (16). In addition to *Porphyromonas gingivalis*, other pathogenic bacteria can trigger destructive inflammation involving both innate and adaptive immune factors (61). Host oral tissue immunoreaction further induces inflammation, thereby causing irreversible downstream pathological changes. Mice with oral mucosal damage that underwent OMT from healthy mice exhibited a reconstruction of the epithelium and tongue papillae, decreased leukocyte infiltration in the oral epithelium, and amelioration of oral mucositis, thereby demonstrating the causal effects of the oral microbiota on local tissues (62).

**Figure 2:**
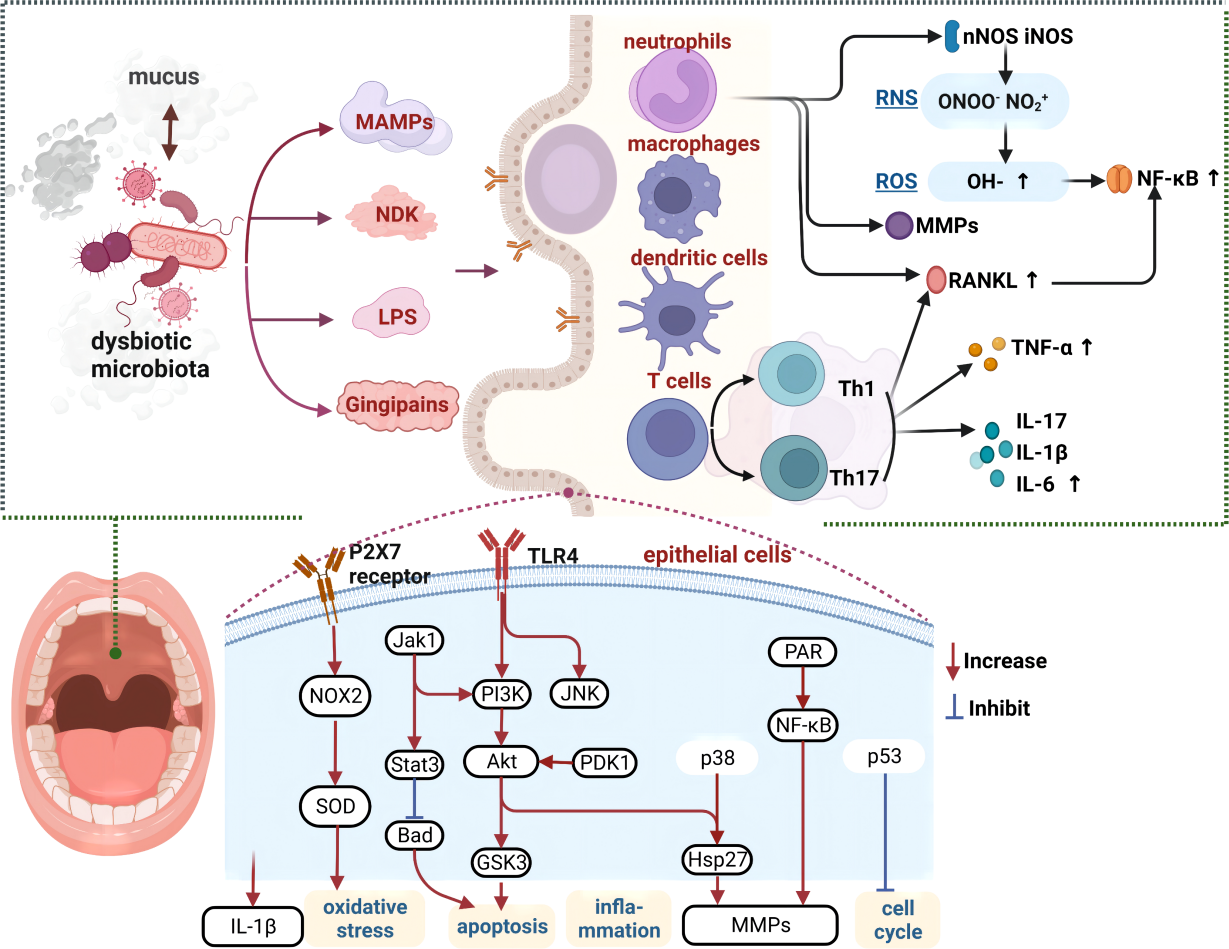
Crosstalk mechanisms between the oral microbiota and local tissues Crosstalk of the oral microbiota with saliva and oral epithelial tissues, causing epithelial cell apoptosis, immune cell proliferation, and inflammation, which may further exacerbate dysbiosis. Various cytokines act on vascular endothelial cells to facilitate the onset of cardiovascular disease. MAMPs, microbe-associated molecular patterns; NDK, nucleoside-diphosphate-kinases; LPS, lipopolysaccharide; TLR, toll-like receptors; MMPs, matrix metalloproteinases; ROS, reactive oxygen species; RNS, reactive nitrogen species; TNF-α, tumor necrosis factor-α; MMPs, matrix metalloproteinases; Th, T helper; IL, interleukins.

The oral microbiota also interacts with mucus such as saliva. Moreover, mucus rinses microorganisms off from the inner epithelial surface, while also establishing a protective barrier between the microbiota and the oral epithelium (63). Oral mucus is mainly composed of mucins, which are densely glycosylated polymers that can form three-dimensional structures (64). The oral soft tissues and teeth are coated by a thin film predominantly composed of mucins such as MUC5B and MUC7. Mucins contain glycans that serve as the main energy source for the oral microbiota. Many microorganisms contain genes encoding the relevant enzymes that break down and digest these glycans. Mucus also affects the competition between the microbiota, and hence the viability of bacterial species. For example, previous studies using an artificial model of salivary mucins reported that mucins promote the co-existence of two competing bacterial species, *Streptococcus mutans* and *Streptococcus sanguinis*. Furthermore, other studies have confirmed that mucins prevent the formation of biofilms by pathogens, including *Streptococcus mutans (* 65, 66). MUC5B affects intraspecific interactions by promoting the production of bacterial proteomes. For example, *Streptococcus gordonii* cultured with MUC5B promoted the production of six novel biofilm cell proteins and three planktonic proteins, thereby eliciting specific responses in the bacterial biofilm cell proteome (67). These findings highlight the important roles oral environment on the microbiota. **(** **Figure 2** **).**

#### 2.2.2 Bacteremia

Dysbiosis involves microbial invasion of the bloodstream. It is usually transient due to the rapid immune response that follows. Due to the unstable duration and nature of bacteremia (68), it is hard to confirm that oral microbiota is directly involved in the formation of atherosclerotic plaques (69). Similar findings have been reported in gut microbiota research, but a clear link between dysbiosis and cardiovascular disease occurrence has not yet been established (70). Bacteremia may result from local tissue barrier damage. Although the bacterial species found in atherosclerotic plaque formation are common in the oral cavity, they are few (71) **(** **Figure 1** **).** At the species level, *Streptococcus* spp., “red complex” bacteria*, Aggregatibacter actinomycetemcomitans, Fusobacterium nucleatum*, and *Prevotella intermedia* have been detected in atherosclerotic plaques (10, 11, 72). The antibodies induced by *Porphyromonas gingivalis* are commonly found in saliva and subgingival sites (60, 72). The cross-reactivity of bacterial antibodies (predominantly against *Firmicutes, Pseudomonadota*, and *Bacteroidetes*) with human heat shock protein 60 in host endothelial cells can elicit autoimmune responses, thereby activating a series of cascade reactions and promoting atherosclerosis (73). Bacterial surface proteins, such as glycosyltransferase, the sialic acid-binding human serum albumin (Hsa), and the co-aggregation proteins CshA and CshB, play a key role in host endothelial cell invasion and bacteremia development (51). Some bacterial surface proteins bind to host pattern recognition receptors. For example, bacterial surface proteins in the bloodstream that bind to nucleotide-binding oligomerization domain 2 receptors in the brain can regulate metabolism, body temperature, and appetite (74). In addition, bacterial structures, such as the flagellum, may destroy tissue barriers by acting as virulence factors, thereby increasing inflammation (75).

The microbiota dysbiosis could promote bone marrow activity and increase risk of cardiovascular diseases. Cytokines and bacterial by-products are known to increase marrow myelopoiesis and glycolysis *via* the hematopoietic-arterial axis or inflamed periodontal tissues which aggravate arterial inflammation (76).

#### 2.2.3 Oral-gut microbiota transfer

Studies have found that there may be an overlap of approximately 45% between the oral and gut microbiotas (77), and the oral microbiota may lead to gut dysbiosis *via* oral-gut microbiota transfer or through other pathways **(** **Figure 1** **)**. Similarities can be detected between the microbial colonization of the oral and gut microbiotas (78). Furthermore, certain genetic variations in the host are associated with both types of microbiotas, as demonstrated by the identification of five genetic loci that were significantly associated with the oral microbiota, three of which were also significantly associated with the gut microbiota (79). The oral microbiota is mainly composed of five main phyla (*Protebacteria, Firmicutes, Bacteroidetes, Actinobacteria, and Fusobacteriota*) (7), of which *Proteobacteria; Neisseria*, *Firmicutes and Streptococcus* are the most extensively studied. However, studies have found that the dominant genus in the oral cavity may differ between people in different countries (Chinese population was dominated by *Neisseria (* 7), Canadian population was dominated by *Veillonella (* 47), Qatari population was dominated by *Prevotella (* 48)), which may be related to sociodemographics. The gut microbiota generally consists of six main phyla (*Firmicutes, Bacteroidetes, Actinobacteria, Pseudomonadota, Fusobacteriota*, and *Verrucomicrobiota*), of which Firmicutes and Bacteroidetes are the dominant phyla (80). Hitherto, the modes of oral-gut interactions remain poorly understood.

The majority of existing studies involve orally administering mice with oral microbiota and observing changes in the composition of gut microbiota. A previous study reported that an oral administration of *Porphyromonas gingivalis* triggers a clear floristic separation in gut microbiota, with a significant increase and decrease in the proportions of Bacteroidetes and Firmicutes, respectively. This was accompanied by a decreased mRNA expression of tight junction proteins (TJPs) in the small intestines and a downregulated genetic expression of TJP-1 and occludin, which are involved in intestinal permeability, thus leading to increased intestinal permeability and impaired barrier function (53). In addition to altering immunomodulation and gut barrier function, the oral administration of *Porphyromonas gingivalis* can affect host metabolism. Mounting evidence suggests that alterations in gut microbiota underlie the pathology of metabolic diseases *via* gut metabolite profile modulation (53, 61). The oral administration of *Porphyromonas gingivalis* in C57BL/6 mice decreased and increased the relative abundances of *Bacteroidetes* and *Deferribacterota* in the gut microbiota, respectively. Moreover, Kyoto Encyclopedia of Genes and Genomes (KEGG) analysis revealed significant decreases in the activation of pathways related to amino and nucleotide sugar metabolisms, chaperones and folding catalysts, glycosyltransferases, limonene, and pinene degradation, as well as folate biosynthesis (81). Interestingly, the oral administration of *Porphyromonas gingivalis* had opposite effects on the relative abundances of gut *Bacteroidetes* and *Firmicutes* in both studies. Moreover, the proportions of gut *Bacteroidetes* and *Firmicutes* are key biomarkers in patients with cardiovascular disease (such as hypertension, coronary heart disease, and stroke), which also decrease and increase the relative abundances of *Bacteroidetes* and *Firmicutes*, respectively (82–84). In a large-scale study of salivary and fecal microbiota in individuals from five countries, bioinformatic analysis revealed that 10% of the oral microbiota are transferred to and subsequently colonize the gut (52). It has been found that the species transfer of opportunistic pathogens is more frequent among diseased individuals; however, the presence of *Fusobacterium nucleatum* subspecies may facilitate this transfer, and therefore aggravate disease severity (85).

OMT can be employed to further investigate the effect of oral microbiota on the composition of gut microbiota. Following the oral transplantation of *Fusobacterium nucleatum* in healthy mice, an elevated conversion of protein 1 light chain 3-I (LC3-I) to protein 1 light chain 3-II (LC3-II) was observed in the colorectal tissue (LC3-II is an important molecular marker of autophagy), and the administration of antibiotics such as metronidazole eliminated this phenomenon. In addition, *Fusobacterium nucleatum* transplantation led to changes in fecal microbiota composition, as demonstrated by an increased abundance of fecal *Fusobacterium nucleatum* (85). Thus, we can assume that the gut microbiota affects the progression of oral mucositis, whereas OMT reduces the malignant reduction of oral and gut bacteria and regulates the gene expression of lingual tissues, and hence OMT has potential therapeutic significance (62). The abovementioned two studies illustrate the causality behind oral-gut microbiota transfer. However, there is ongoing debate as to whether the effects of the oral microbiota are predominantly pathogenic or therapeutic, and further investigations are needed on cardiovascular models.

### 2.3 Host genotype affects the oral microbiota

Metagenome-genome-wide association studies have revealed that host genes promote the growth of specific oral bacteria. Leucine zipper motif isoform 2 (APPL2) and glucose transporter 9 (SLC2A9) gene polymorphisms have been shown to affect the abundances of multiple oral bacteria and fecal *Bifidobacterium animalis*. These metabolism-related genes are closely associated with obesity and insulin resistance; further, the mechanisms by which these gene polymorphisms affect bacterial abundance may involve specific oral bacterial growth regulation through host microRNAs (79, 86). Loci CAMTA1 (intron variant)/VAMP3 (rs1616122) (p<5 ×10^-6^) (87) and loci VAMP8 (rs1561198) (p<5 ×10^-8^) (88) may be replicated in the genetic risk locus of cardiovascular diseases and periodontitis. The VAMP8 function is related to membrane vesicular trafficking and corrupting host immune defense (88). The long non-coding RNA ANRIL (antisense noncoding RNA in the INK4 locus) regulates glucose and fatty acid metabolism and is associated with periodontitis (89).

In addition, epigenetic mechanisms can affect the microbiota (90). Obesity, insulin resistance, and angiogenic responses are all cardiovascular risk factors that are closely related to epigenetic mechanisms, which may promote pathogenesis. In a pathological state, host immunodeficiency may shift the balance towards dysbiosis, thereby transforming commensals into pro-inflammatory pathobionts (39).

## 3 The oral microbiota is involved in the formation of products related to energy metabolism

Previous studies have mostly focused on the identification of microbial communities that are related to cardiovascular event occurrence, and less on oral microbiota metabolites. The oral cavity is also responsible for the metabolism of energy substances, and hence the effects of metabolites produced by the oral microbiota on the host should not be overlooked (91). As with the gut microbiota, the oral microbiota contains a large number (approximately 1839) of biosynthetic gene clusters that produce a variety of metabolites *via* a wide range of mechanisms (78). Currently, bacteriocins and sactipeptides are the most popular secondary metabolites in oral microbiota research, although their actions have not been linked to cardiovascular disease occurrence. This paper mainly introduces the metabolites that are closely associated with cardiovascular disease occurrence and examines the differences between oral and gut microbiotas.

### 3.1 Short-chain fatty acids

SCFAs are key metabolites produced by the microbiota that are involved in the host’s inflammatory response, lipid metabolism pathway, and gluconeogenesis (92, 93). Pyruvate is produced by the microbiota *via* glycolysis and the pentose phosphate pathway, and then converted *via* other branch pathways into SCFAs, such as acetic, propionic, butyric, and isobutyric acids (94). Amino acids can also be metabolized to produce small amounts of SCFAs (91). Oral bacteria can utilize carbohydrate-active enzymes for the degradation of carbohydrates into SCFAs, which then support their own energy metabolism (95). The proteases and peptidases produced by the microbiota break down proteins in food, and the resulting peptides and amino acids are converted into SCFAs after deamination (96, 97). Therefore, different dietary habits, especially sugar intake, can immensely affect the oral microbiota (96–98). Bacteria that are capable of utilizing sugars to produce SCFAs include *Streptococcus, Actinomyces, Lactobacillus*, *Propionibacterium*, and *Prevotella (* 91, 99).

There is conflicting evidence surrounding the local and systemic effects of SCFAs in the oral cavity. On the one hand, whilst breaking down carbohydrates to produce SCFAs, the oral microbiota also generates lactic and acetic acids, thereby causing SCFA-producing bacteria to act as a double-edged sword. Lactic and acetic acids reduce the local pH, leading to dysbiosis development, which exacerbates periodontitis and dental caries (98, 100). Lactic acid may also promote immune cell activation and damage oral epithelial cells, leading to persistent local inflammation (101). On the other hand, there is an ongoing debate as to whether SCFAs exert protective or destructive effects on the oral cavity. Due to the differences in host tissues, gut SCFAs may reduce the occurrence of intestinal epithelial cell apoptosis and autophagy *via* the phosphatidylinositide 3-kinases/protein kinase B/mammalian target of rapamycin (PI3K/Akt/mTOR) pathway, which leads to the protection of the local mucosal barrier (102, 103); nevertheless, oral SCFAs have been found to alter the expressions of connexins and adhesion proteins, thereby impairing oral epithelial cell function (104). Pathogenic bacteria that cause tumor proliferation and metastasis (105) generally exhibit glycolysis and acid production functions, which decrease the pH and cause dysbiosis. Examples of such bacteria include *Bifidobacterium longum, Bifidobacterium dentium, Streptococcus mutans*, and *Scardovia wiggsiae* (98, 106, 107).

**Figure 3:**
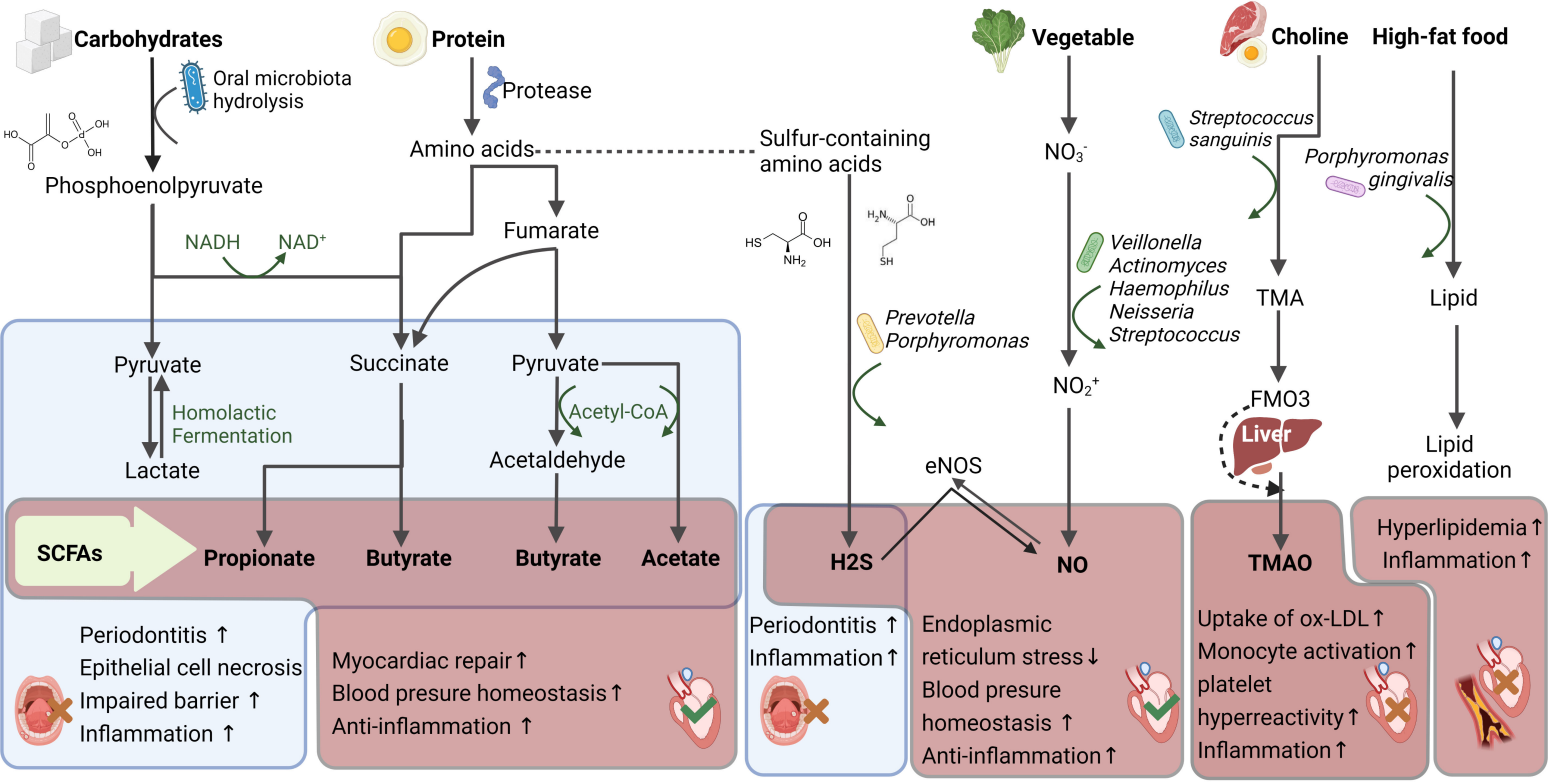
Oral microbiota metabolites and its impact upon cardiovascular health. The oral microbiota produce short chain fatty acids (SCFAs), hydrogen sulfide (H_2_S), nitric oxide (NO), trimethylamine oxide (TMAO), lipids and other metabolites from the digestion of different foods, which affect oral and cardiovascular health. The red box displays the cardiovascular effects of the metabolites and the blue displays the oral effects. Red wrong number refers to harmful, while green right number refers to beneficial. DANH/NAD+, Nicotinamide adenine dinucleotide; ox-LDL, Oxidized-Low-Density Lipoprotein Cholesterol; Flavin-containing monooxygenase 3, FMAO3; Trimethylamine, TMA.

SCFAs exhibit anti-inflammatory effects in plasma. Mice supplemented with 1% butyrate for 10 weeks displayed a 50% reduction in the area of aortic plaques compared with those in the control group; this suggests that butyrate may have an anti-inflammatory function (84). SCFAs are also known to inhibit the NF-κB and Akt signaling pathways, thereby reducing plasma cytokine (TNF-α, IL-12, and INF-γ) levels to achieve anti-inflammatory effects and increase peroxisome proliferator-activated receptor-γ (PPAR-γ) pathway expression to improve insulin sensitivity (108, 109). Furthermore, SCFAs suppress histone deacetylases (HDACs) and bind with specific G protein-coupled receptors (GPRs) to achieve cardiovascular protective effects (110). SCFAs that act as HDAC inhibitors include valproic acid and sodium butyrate; moreover, the reversible lysine acetylation process is closely associated with myocardial infarct size reduction, myocardial hypertrophy, and cardiac fibrosis suppressions, as well as angiogenesis promotion (111, 112). In spontaneously hypertensive rats, HDAC activation was found to promote hypertension and myocardial hypertrophy occurrence, whereas valproic acid administration led to the reversion of inflammation and hypertension reversions (113, 114). Among the GPRs, GPR43 is expressed in the heart and binds with a wide range of SCFAs from formic to valeric acid, to enhance insulin sensitivity, energy expenditure, and anti-inflammatory effects (115). Furthermore, Olfactory receptor78 (Olfr78) and GPR41, which are expressed in the kidneys, can facilitate blood pressure reduction in response to propionate administration (116). More specifically, Olfr78 is expressed in the juxtaglomerular apparatus to mediate renin secretion (117), and GPR41 is expressed in the smooth muscle cells of renal blood vessels to reduce vascular resistance (116, 118). Therefore, SCFAs are promising research target metabolites, and the extent to which they are involved in cardiovascular disease processes warrants further exploration (**Figure 3**).

### 3.2 Nitric oxide

Nitric oxide (NO) is an important gaseous signaling molecule involved in endo- and exogenous metabolic pathways (119), and the oral microbiota are a key NO source for exogenous metabolism. When there is insufficient NO synthesis mediated by endogenous nitric oxide synthase (NOS), the nitrite (NO_2_^-^) produced by the oral microbiota serves as an important NO reservoir in the bloodstream and tissues. Thus, the absence of specific nitrate (NO_3_^-^)-reducing bacteria or alterations in oral microbiota structure may disrupt the NO_3_^–^NO_2_^–^NO pathway, leading to NO insufficiency in the body (120). Dietary NO_3_^-^ is mainly sourced from green leafy vegetables and can be reduced by the oral microbiota to NO_2_^-^ and NO, *via* bacterial NO_3_^-^ reductases. As humans lack these enzymes, this process cannot be accomplished by the host alone. Dietary NO_3_^-^ is recycled in the human body through enterosalivary NO_3_^-^ circulation. NO_3_^-^ is first absorbed in the proximal digestive tract along with food; approximately 25% of NO_3_^-^ is actively concentrated in the salivary glands, such that NO_3_^-^ concentration in saliva is 20 times higher than that in plasma (121, 122). Following concentration in the salivary glands, salivary NO_3_^-^ is reduced *via* the action of NOS and the anaerobic pathway to form NO_2_^-^, which re-enters the body through mastication. Subsequently, NO_2_^-^ is converted to nitrous acid in the digestive tract, followed by the formation of NO and NO donors, as well as a series of secondary nitrosation and nitrification products from reactions facilitated by the low gastric pH (123), or further reduction by nitrite reductases released by the gut microbiota (124). The genera *Veillonella, Actinomyces, Haemophilus, Neisseria, Streptococcus* have been reported to exhibit NO_3_^-^ reduction function (125, 126).

Both NO_2_^-^ and NO have strong NO signaling effects that can stimulate the circulatory system to promote systemic health (127). NO_2_^-^ metabolism is activated by hypoxia, low pH, and reactions with metalloproteins (128, 129). NO is a key molecule in the oxidative stress pathway with vasoactive and endothelial protective effects. It can reduce blood pressure, ameliorate atherosclerosis, protect against ischemia-reperfusion injury, improve platelet aggregation, and exert anti-inflammatory effects (129). NO deficiency is closely associated with cardiovascular disease onset, and therefore NO can serve as a predictor of cardiovascular events (130). The level of NO produced by the oral microbiota affects the cardiovascular disease course. Using a murine periodontitis model, a previous study found that infection with *Porphyromonas gingivalis, Treponema denticola*, and *Tannerella forsythia* for 16 weeks led to significant plasma NO level reduction, NO-related pathway (tetrahydrobiopterin/neuronal nitric oxide synthase/Nuclear factor (erythroid-derived 2)-like 2 (BH4/nNOS/NRF2)) inhibition in the colon and plasma, and atherosclerotic plaque area increase. Therefore, oral dysbiosis triggered a reduction in NO synthesis and bioavailability, which resulted in impaired vascular function (131). In ApoE^-/-^ mice colonized with *Porphyromonas gingivalis, Treponema denticola, Tannerella forsythia*, and *Fusobacterium nucleatum* for 24 weeks, a significant decrease in plasma NO levels was observed, which was accompanied by significant increases in the levels of inflammatory factors such as IL-1β, IL-13, IL-4, lymphotactin, and the regulated chemokine (upon on activation normal T cell expressed and secreted factor (RANTES) (132)). Furthermore, the mice developed bacteremia, inflammatory response, and atherosclerosis. These findings indicate that NO, which is mediated by the oral and gut microbiotas, also plays a crucial role in the pathogenesis of atherosclerosis (**Figure 3**).

### 3.3 Hydrogen sulfide

The oral environment is rich in sulfur-containing amino acids (such as cysteine, and methionine) that can be metabolized *via* proteolytic bacteria (including *Prevotella* and *Porphyromonas*) to produce hydrogen sulfide (H_2_S). H_2_S is considered the third most important endogenous gaseous signaling molecule, after CO and NO, and plays a physiological role in life processes. Its metabolic process in the body mainly involves the use of L-cysteine and L-homocysteine as substrates and is completed under enzymatic catalysis. A paradox also exists for H_2_S; the presence of H_2_S in the oral cavity may lead to halitosis (133), enhance oral inflammation (134), and even increase the risk of oral cancer (135). However, in plasma, H_2_S triggers strong anti-oxidation, anti-inflammation, as well as insulin resistance improvement, and thus can regulate several cardiovascular functions (136). Furthermore, H_2_S may exert more beneficial effects by interacting with NO. For example, H_2_S can activate endothelial NOS *via* the phosphorylation of Ser1177, which significantly increases the bioavailability of NO and NO-mediated cytoprotective signaling (137).

H_2_S is produced by a minority of oral bacteria, and hence remains at low levels in healthy individuals; however, an elevated oral microbial load associated with the oral disease can significantly increase overall H_2_S levels (138), which amplifies the inflammatory response, thereby leading to periodontal disease onset. One possible explanation for the amplified inflammatory response is that H_2_S can trigger the release of IL-1β and IL-18, which are inflammatory cytokines. A previous study demonstrated that the dose-dependent activation of the cyclooxygenase-2 (COX-2), Akt, and extracellular regulated protein kinases1/2 (ERK1/2) pathways by H_2_S can promote the proliferation of oral cancer cells (135). It is currently unclear whether the H_2_S produced by the oral microbiota can affect plasma H_2_S concentration and the related metabolic pathways. However, existing studies have confirmed that the oral administration of H_2_S may have a beneficial effect on cardiovascular metabolism (139, 140). Dietary supplementation with garlic oil (a natural source of H_2_S) can help to increase renal mRNA expression, H_2_S-generating enzyme activity, NO bioavailability, and plasma SCFA levels. Moreover, garlic oil supplementation during lactation and pregnancy reportedly confers protection against hypertension in adult offspring (141). In addition, the oral administration of sulfur-containing products can restore the circulating levels of sulfides, and H_2_S therapy has been found to restore adiponectin levels and suppress high-fat diet (HFD)-induced cardiac endoplasmic reticulum stress. It has been reported that intraperitoneal injection of Na_2_S improves survival through attenuation of inflammasome-mediated adverse remodeling (142). Furthermore, plasma and myocardial H_2_S levels play important roles in the pathophysiology of diabetic cardiomyopathy (143). However, oral bacteria produce very low concentrations of H_2_S, and there is no evidence of simultaneous changes in H_2_S metabolism in oral and cardiac tissues (**Figure 3**).

### 3.4 Lipid metabolites

A HFD induces significant changes in the oral microbiota (144); in addition, it causes lipid regulator activity elevation or atherosclerosis-promoting metabolite production through specific bacterial populations. For example, trimethylamine oxide (TMAO) accelerates cardiac remodeling, stimulates the renin-angiotensin system, increases oxidative stress, and accelerates endothelial dysfunction, which can promote the development of cardiovascular diseases such as heart failure, hypertension, coronary heart disease, and arrhythmia. The gut microbiota has been thought to be the main source of TMAO (145). Specifically, the gut microbiota converts choline and carnitine from ingested meat and eggs into trimethylamine (TMA), which passively diffuses into the bloodstream through the intestinal wall and enters *via* the portal vein into the liver, where it is oxidized by flavin-containing monooxygenases into TMAO. In the oral cavity, TMAO can also be produced by *Streptococcus sanguinis*, which has been shown to enhance the role of the gut microbiota in TMA-accelerated atherosclerosis (146). Furthermore, oral dysbiosis may exacerbate dyslipidemia and *Porphyromonas gingivalis* has been found to have a significant proteolytic effect on (6)lipoproteins and is involved in the aggravation of lipid peroxidation (147) (**Figure 3**).

## 4 Oral microbiota transplantation facilitates research on systemic diseases

Three issues are still heavily debated: 1) the causal relationship between oral microbiota and cardiovascular diseases; 2) the pathological mechanism of oral dysbiosis rather than periodontal disease; 3) the therapeutic effect of oral microbiota. In terms of treatment, dietary supplements, such as arginine, can also substantially affect the composition and metabolic output of oral microbial communities and are known to be involved in NO regulation (148, 149). Microbiota sequencing suggests that brushing the teeth could not only remove dental plaque but may also have a positive effect on the oral chemical environment and the metabolism of the oral microbial population (150). The combined analysis of multi-omics and the support of experimental techniques are improving the current situation.

**Figure 4:**
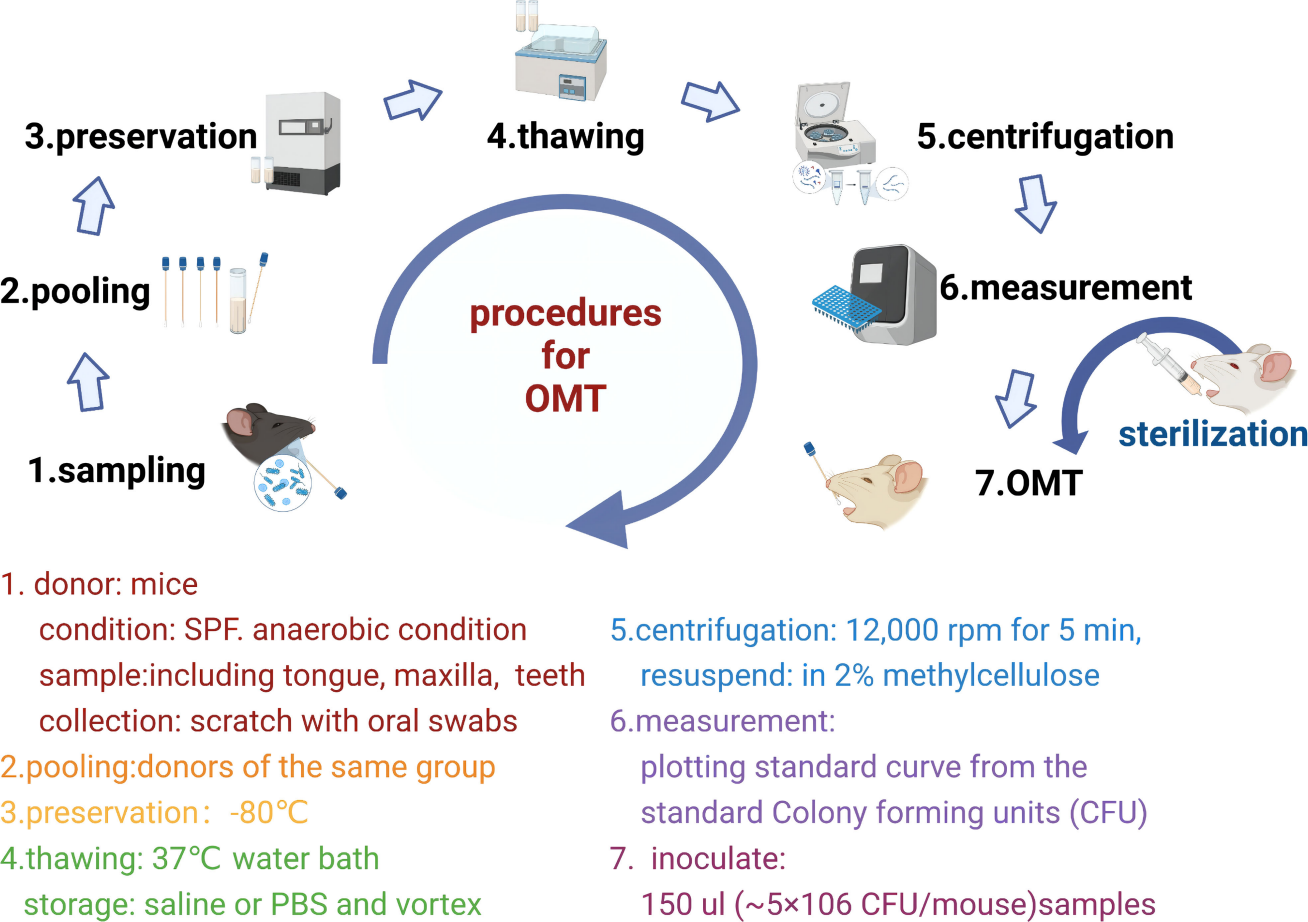
Flowchart of a standardized oral microbiota transplantation protocol.

OMT and animal models that mimic the oral dysbiosis of humans could be used to reconstruct the oral microbiota in mice and observe changes in cardiovascular disease phenotypes. It has been reported that oral microbiota may influence the composition of the intestinal microbiota and induce intestinal injury after transplantation. Oral *Fusobacterium nucleatum* infection is reportedly an exacerbating factor of colon cancer and affects the efficacy of radiotherapy (85). In terms of treatment, OMT ameliorates oral mucositis, which manifests as a remodeling of the oral mucosal epithelium and lingual papillae, a decrease in the leukocyte count, and an increase in the number of proliferating oral epithelial cells (62). Standardized sampling strategies (151)and sterilization of oral microbiota (152) are the basis of OMT. At present, few animal-based studies have employed the OMT technique; nevertheless, a consensus has not been reached with regard to its protocol, and thus further investigation is warranted. Based on the literature, we have compiled a standardized OMT protocol for the mechanism of cardiovascular disease in the future (62, 85, 153) **(** **Figure 4** **)**.

Sample preparation: Aimed at human systemic diseases, sampling sites include saliva, supragingival plaque, subgingival plaque, tongue coating, etc (151). In animal experiments, due to the limitation of the oral region, the whole oral cavity is generally collected (152).

Recipient mice preparation: It’s not mandatory to deplete the oral microbiota of recipient mice. To deplete specific bacteria, fluoroquinolones are generally more effective against gram-negative bacteria compared to gram-positive bacteria (152). Metronidazole can remove gram-negative bacteria (85).

## 5 Discussion

In 2021, *Nature* specifically launched research on special topics in oral health, and repeatedly proposed the potential research value of the oral microbiota and its relationship with chronic diseases (154, 155). There is mounting evidence to support the crucial role of oral microbiota in cardiometabolic health and diseases. However, the effects of the oral microbiota on oral and cardiovascular health remain paradoxical. A more conclusive finding among the numerous studies is that oral *Porphyromonas gingivalis* and *Porphyromonas endodontalis* can trigger oral and systemic inflammation as well as immunoreaction in the host. Under conditions of oral dysbiosis, chronic inflammation and persistent infection may cause a buildup of immunological memory in immune cells, which elicits an overreaction of the immune system to inflammatory and bacterial signals, thereby creating a mutually reinforcing vicious cycle. The oral microbiota causes local barrier damage and bacteremia, which have been previously demonstrated in the gut microbiota. However, the oral microbiota is upstream to the digestive tract, and therefore can affect the gut microbiota *via* microbial transplantation, thereby further aggravating the cardiovascular disease. This may be a pathogenic mechanism that is unique to the oral microbiota. Furthermore, the oral microbiota is involved in various forms of energy metabolism. Carbohydrates and proteins are metabolized by the oral microbiota to produce SCFAs. Plant-based dietary NO_3_^-^ are metabolized to produce NO, which has vasoactive effects. Aromatic and sulfur-containing amino acids produce indole and H_2_S, respectively; moreover, H_2_S causes vascular smooth muscle relaxation and therefore confers cardiovascular protection. The oral microbiota also intervenes in the HFD-induced elevation of blood lipids. Hence, the oral microbiota is a catalyst for the development of cardiovascular disease induced by poor dietary habits.

The metabolites of the oral microbiota have not yet been fully explored. Although it is known that microbiota structure, LPS, and metabolites directly affect an individual’s health, there is no consensus concerning which of the factors is the predominant pathogenic factor. The effects and mechanisms of secondary gut microbiota metabolites (such as bile acids and TMAO) on cardiovascular disease occurrence have been verified, although these effects have not been detected using secondary oral microbiota metabolites. Despite ample research demonstrating the potential significance of oral microbiota, no study has reported a direct link between oral microbiota and cardiovascular disease occurrence. Numerous questions remain unanswered concerning the abovementioned relationship from the perspective of microbiota metabolites or composition. Many microbes in the oral cavity are dependent on commensals, and thus cannot be cultured alone, which may pose obstacles to understanding the functions of the microbiota. To overcome this limitation and evaluate the effects of the oral microbiota on cardiovascular disease occurrence, a future direction of research should be aimed at developing culture-free deep metagenomic sequencing and single-cell sequencing techniques. Interestingly, gut microbiota research may provide numerous ideas and methods for reference in oral microbiota research. Studies have shown that the oral microbiota can be modulated using mouthwash (156) or vegetable-derived nitrate (157, 158) hence carrying out cardioprotective effects, while tooth brushing and periodontal therapy can somewhat ameliorate the severity of cardiovascular diseases (159–162). However, there is currently a lack of targeted therapy for the oral microbiota, and dietary interventions are still in the preliminary stage.

Traditional Chinese medicine theorizes that the human tongue coating can assist in the diagnosis and treatment of diseases. This clinical practice has been verified at the microbiome level, and the presence of *Campylobacter concisus* in the tongue coating can be used to guide the early diagnosis of gastric cancer (163). There is a traditional Chinese medicinal theory that “the tongue is the window of the heart”. Thus, an accurate analysis of the composition and function of the oral microbiota will contribute to the diagnosis and treatment of cardiovascular disease. This may be one of the most perceptive insights for oral microbiota research derived from traditional Chinese medicine and may yield brilliant results in the future.

## Author contributions

YWL and MZ performed the reference collection, conducted the reference analysis, and wrote the manuscript, thus are considered as co-first authors. YL contributed to the topic conception, manuscript revision, and decision to submit for publication. YFL, JC and BL contributed to reference analysis and helped in the revision of the manuscript. All authors contributed to the article and approved the submitted version.

## Funding

This work was supported by the National Natural Science Foundation of China (82022076).

## Acknowledgments

We would like to acknowledge the support of colleagues/students who have contributed to the work relevant to this article and thank Editage (www.editage.cn) for English language editing.

## Conflict of interest

The authors declare that the research was conducted in the absence of any commercial or financial relationships that could be construed as a potential conflict of interest.

## Publisher’s note

All claims expressed in this article are solely those of the authors and do not necessarily represent those of their affiliated organizations, or those of the publisher, the editors and the reviewers. Any product that may be evaluated in this article, or claim that may be made by its manufacturer, is not guaranteed or endorsed by the publisher.
